# Does Methyl Jasmonate Effectively Protect Plants under Heavy Metal Contamination? Fatty Acid Content in Wheat Leaves Exposed to Cadmium with or without Exogenous Methyl Jasmonate Application

**DOI:** 10.3390/biom13040582

**Published:** 2023-03-23

**Authors:** Natalia Repkina, Svetlana A. Murzina, Viktor P. Voronin, Natalia Kaznina

**Affiliations:** Institute of Biology of the Karelian Research Centre of the Russian Academy of Sciences (IB KarRC RAS), Petrozavodsk 185910, Russia; murzina.svetlana@gmail.com (S.A.M.); voronen-viktor@mail.ru (V.P.V.); kaznina@krc.karelia.ru (N.K.)

**Keywords:** *Triticum aestivum* L., lipids, heavy metals, jasmonate, adaptation

## Abstract

The effect of methyl jasmonate (MJ) (1 µM) on wheat (*Triticum aestivum* L. cv. Moskovskaya 39), seedlings and the fatty acid (FA) content of leaves under optimal and cadmium (Cd) (100 µM) stress conditions wasinvestigated. Height and biomass accumulation was studied traditionally; the netphotosynthesis rate (P_n_) was studied using a photosynthesis system, FAs’profile—GS-MS. No effect on the height and P_n_ rate of the MJ pre-treatment wheat at optimum growth conditions was found. MJ pre-treatment led to a decrease in the total amount of saturated (about 11%) and unsaturated (about 17%) identified FAs, except α-linoleic FA (ALA), which is probably associated with its involvement in energy-dependent processes. Under Cd impact, the MJ-treated plants had a higher biomass accumulation and P_n_ rate compared to untreated seedlings. Both MJ and Cd caused stress-induced elevation of palmitic acid (PA) versus an absence of myristic acid (MA), which is used for elongation. It is suggested that PA participates in alternative adaptation mechanisms (not only as a constituent of the lipid bilayer of biomembrane) of plants under stress. Overall, the dynamics of FAs showed an increase in the saturated FA that is important in the packing of the biomembrane. It is supposed that the positive effect of MJ is associated with lower Cd content in plants and a higher ALA content in leaves.

## 1. Introduction

Heavy metal contamination hasvariousharmful effects on plants [1,2]. Some elements are toxic only at high concentrations; low concentrations of them are vital for plants. There are also elements that have a negative effect even at low concentrations; their physiological role is often unknown [3]. Hazardous heavy metals are linked to their capability to accumulate in plants, with further transit through the food-intake chain to animals and humans, causing a negative effect on health [4]. In that context, the problem of environmental pollution by heavy metals is still a major environmental concern.

Cadmium (Cd) is one of the most toxic heavy metals. Even at low concentrations it can cause damage at different levels of a structural organization [5]. The main sources of its increasing concentration in the environment are divided into natural factors (weathering of parent rocks, volcanic activity) and anthropogenic factors (including mining, chemical and heavy industries, the excessive application of mineral fertilizers, etc.) [6]. Cd in low concentrations has a strong negative effect on plants. However, plant organisms have a whole range of defense mechanisms that operate at different levels of the organization, which are aimed at preventing the absorption of the metal and its inactivation in the cells. Among them is the activation of the components of the antioxidant system, which is necessary to prevent the accumulation of excess reactive oxygen species and oxidative stress, as well as to increase the activity of enzymes involved in the biosynthesis of low-molecular-weight chelators, including glutathione and its derivatives, capable of binding cadmium ions and isolating them in the vacuole [7]. Currently, the mechanisms of plant adaptation to cadmium continue to be actively studied.

In particular, the effect of various growth regulators and their applications in plants’ tolerance of cadmium has been widely investigated. One of the least studied regulators in this respect is jasmonic acid and its derivatives [8]. Jasmonates are plant phytohormones synthesized from α-linolenic acid via the octadecanoid pathway [9]. They play an important role in plant development, and are also involved in plant responses to various stressors [10,11]. The role of jasmonates in plant resistance to biotic stresses has been studied quite extensively; however, less is known about their role in plant resistance to abiotic factors. There are fragmentary data describing the effect of exogenous applications of jasmonates on plants’ tolerance of heavy metals. Previous research has demonstrated that the application of MJ stimulated the activity of several antioxidant enzymes responsible for reactive oxygen species’ elimination under heavy metals in rice [12], *Brassica napus* [13] and *Solanum lycopersicum* [14]. An increase in soluble sugars and osmolyte content by jasmonic acid application in plants exposed to cadmium was also shown [15,16]. Exogenous jasmonates’ application caused a decrease in the MDA level in *B. napus*, *V. faba* and rice that also supported a positive effect on a decrease in oxidative damage at heavy metals influence [12,13,16]. Along with antioxidant system promotion, MJ has been suggested as being involved in thiol metabolism, being particularly increased in gene expression, encoding enzymes of glutathione biosynthesis and causing an increased reduction in glutathione content and a positive effect on sulfur assimilation; all of these are necessary for the effective binding and inactivation of metal ions [17]. MJ was also able to protect the photosynthetic apparatus from heavy metal stress through the increase of pigment content, the total rate of photosynthesis and PSII efficiency [17,18]. Exogenous MJ treatment caused an increase in the endogenous MJ level [19]. Moreover, it was shown that the pre-treatment of seeds with methyl jasmonate (MJ) leads to a decrease in heavy metal accumulation by plants [20], but the mechanisms that are the basis of this are still unknown. As a result of the facts presented above, it is possible to conclude that jasmonates play a significant role in plant adaptation to heavy metals. Nevertheless, there are fewer data about the effects of exogenous methyl jasmonate on FA composition changes in plants exposed to cadmium.

It is well known that quantity and quality changes in the FA profile and lipid content, aimed at maintaining the structural integrity and functionality of cell membranes, play an important role in plant adaptations to various stresses [21]. Apart from their structural function, fatty acids play an important role in energy balance and signaling in cells [22,23,24]. They play a key role in the processes of plant growth and development, and also participate in plants’ resistance to various stressors [25]. It should also be taken into account that lipids and fatty acids are important components of the nutritional value of food products. As a result, the changes in their composition under adverse environmental conditions in important agricultural crops, including wheat, are of particular interest.

The aim of this study was to investigate the effect of methyl jasmonate on the fatty acid profile of wheat seedlings exposed to cadmium.

Based on the information presented above and the aim of the study, it was hypothesized that the positive effect of the pre-treatment of wheat seedlings with exogenous methyl jasmonate under the impact of cadmium might be associated with a lower Cd intake by plants in comparison with untreated seedlings; the chemical stimulus (MJ or Cd) might increase in the saturated fatty acids, which can be suggested as an alternative mechanism of plants’ adaptation to chemical stress agents;MJ pre-treatment might lead to achange in the content of the α-linoleic fatty acid that maintains the net-photosynthesis rate and was kept as a source for jasmonate synthesis.

## 2. Materials and Methods

### 2.1. Plant Material and Growth Conditions

Seeds of winter wheat (*Triticum aestivum* L. cv. Moskovskaya 39) from the Tula Research Institute of Agriculture, Russia, were used in the experiment. The seedlings were grown in the growth chamber under the following conditions: temperature 22 °C, relative humidity of air approximately 60–70%, photosynthetic photon flux density 180 µmol m^−2^s^−1^and photoperiod 14 h. These conditions were common for all groups of the experiment.

Design of experiment:

- ‘Control’ seedlings growing in Hoagland solution(pH 6.2–6.4);
- ‘Control + MJ’ 7-day-old seedlings placed in MJ (1 µM) (Sigma-Aldrich, St. Louis, MO, USA) solution for 24 h and then placed back in Hoagland solution for 7 days;
- ‘Cd’ 8-day-old seedlings placed in CdSO_4_ solution (100 µM) for 7 days;
- ‘Cd + MJ’ 7-day-old seedlings placed in MJ (1 µM) (Sigma-Aldrich, St. Louis, MO, USA) solution for 24 h and then placed in CdSO_4_ solution (100 µM) for 7 days.

All measurements were performed on the first leaf of wheat seedlings.

### 2.2. Determination of Cd Content in Wheat Leaves

The Cd concentration in wheat leaves was expressed as mg per kg DW and was analyzed by atomic absorption spectroscopy (AA-7000 Shimadzu, Japan) after prior mineralization in a solution of HNO_3_ and HCl (9:1 *v*/*v*) using Speedwave Digestion (Berghof, Eningen, Germany).

### 2.3. Growth Parameters and Photosynthetic Rate Measurement

The height of the seedlings was measured using the standard method. The 7-day-old wheat seedlings were rinsed with distilled water and blotted with tissue paper for fresh weight (FW) measurements. The net photosynthetic rate (P_n_) was measured on the first fully developed leaves using a portable photosynthesis system HCM-1000 (Walz, Effeltrich, Germany).

### 2.4. Lipid Extraction

The total lipids (TL) from 0.2 g of fresh leaves (mixed sample from 10 leaves fromwheat seedlings) were extracted using the Folch method with a mixture of chloroform–methanol (2:1 *v*/*v*) [26]. In brief, the samples were filtered using a paper filter, Red Ribbon (pore size 5–8μm), with 20 mL of the mixture, after which the precipitate on the filter was washed with 10 mL of pure chloroform. To remove water-soluble impurities, 10 mL of purified deionized water (Simplicity, Millipore, Merck, Darmstadt, Germany) was added to the filtered mixture and left for 2 h in separating funnels (Schott Duran, Mainz, Germany) until the organic phases were completely separated. Lipids remained in the lower chloroform layer, whereas non-lipid substances moved to the upper aqueous methanol phase. Then, the chloroform layer was withdrawn to evaporate under a vacuum on a rotary evaporator—Hei-VAP Advantage HL/G3 (Heidolph, Schwalbach, Germany)—and dried in a vacuum over phosphoric anhydride to a constant weight. The dried samples were re-dissolved with chloroform–methanol (1:1 *v*/*v*) and stored at −20 °C until further processing.

### 2.5. Fatty Acid Analysis

The qualitative and quantitative fatty acid (FA) profile of the TL was analyzed through gas–liquid chromatography (GC) with mass-selective detector (MS); we had previously subjected the TL mixture to acid methylation. To obtain the fatty acid methyl esters (FAMEs) from the TLs, 0.2 mL of a solution of TLs, 2 mL of methanol and 0.2 mL of chlorate acetyl (CH_3_COCl) as catalyst were added to a glass retort (Schott Duran, Mainz, Germany). The obtained solution was heated for 90 min at a fixed temperature of 70–80 °C. After methylation (followed by cooling), 5 mL of the hexane was poured for each sample. For phase separation, 2 mL of deionized water was poured into each glass retort and transferred into the separatory glass funnels for 15 min. FAMEs remained in the upper hexane layer, while residue substances were concentrated in the lower aqueous phase. The hexane layer was evaporated using rotary evaporator Hei-VAP Advantage HL/G3 (Heidolph, Schwabach, Germany) to collect pure FAMEs. Then, 1 mL of hexane for GC (Sigma Aldrich, St. Louis, MO, USA) was added to the collected FAMEs in glass retorts, and the gathered and final solutions were accumulated in GC vials for the following GC analysis.

FAMEs were separated using GC with a mono-quadrupole mass-selective detector: α-Maestro (Saitegra, Moscow, Russia). The separation of FAs was carried out in a gradient thermal configuration (t_start_ = 140 °C—hold 5 min; increase in t from 140 °C to 240 °C at a rate of 4 °C/min; and t_final_ = 240 °C—hold 2 min) with an HP-88 (60 m × 0.25 mm × 0.20 mkm) capillary column (Agilent Technologies, Santa Clara, CA, USA) using helium as a mobile phase. FAMEs were detected in SCAN and SIM modes. The SCAN mode was used for searching and identifying FAs (qualitative analysis) with scan parameters of 50 to 400 m/z. The SIM mode was used for FAs according to analytical standards with Supelco 37 (Sigma Aldrich, St. Louis, MO, USA),with subsequent quantitative analysis of the components that make up the mixture Supelco 37. Quantitative individual FAs in the samples were detected using the calibration method, using Supelco 37 mixture (Sigma Aldrich, St. Louis, MO, USA). The data were analyzed using Maestro Analytic v. 1.026 software(Saitegra, Moscow, Russia) with the NIST library.

### 2.6. Statistical Analysis

All experiments were performed three times with three replicates for each variant (treatments). For measurements of shoot height, fresh weight and net-photosynthesis rate, 15, 10 and 5 biological replicates were performed. The experimental data were expressed as means ± standard errors (SE). An analysis of variance (one-way ANOVA) was used to calculate the least significance difference (LSD) test at *p* ≤ 0.05 to discriminate means. The data were processed using Excel 2007 (Microsoft Corp., Redmond, WA, USA) and analyzed with the Statgraphics Plus 5.0 (Statgraphics Technologies, Inc., The Plains, VA, USA) statistical software.

## 3. Results

### 3.1. Amount of Cd in Wheat Seedling Non- and Pre-Treated by MJ

The Cd concentration in roots and leaves of wheat increased along with the duration of its exposure (Table 1). Moreover, the Cd ions accumulated mostly in roots. Cd concentration in leaves was significantly lower compared to roots (Table 1). The pre-treated wheat seedlings had a lower concentration of cadmium in roots compared to untreated plants. Additionally, the Cd level in leaves was almost twice lower in wheat treated by MJ (Table 1).

### 3.2. Effect of MJ Pre-Treatment on Growth Parameters and Biomass Accumulation

Under optimal growth conditions, the height of MJ-treated plants slightly decreased (Table 2). The effect of cadmium led to a decrease in wheat height on the third day of exposure compared to the control. A greaternegative effect of cadmium on growth was observed on the seventh day of the experiment (Table 1). Pre-treatment by exogenous MJ did not positively affect growth under cadmium stress. Compared with untreated wheat seedlings, the MJ pre-treatment caused a drop in height under cadmium (Table 2).

However, the exogenous MJ application caused an increase in the biomass accumulation of wheat seedlings under normal growth conditions and cadmium stress (Table 3).

### 3.3. Effect of MJ Pre-Treatment on Net Photosynthesis Rate of Wheat Seedlings

The net photosynthesis rate slightly increased after one day of growing inoptimal conditions following a decrease on the third and seventh days (Table 4). In parallel with Cd duration, thenet photosynthesiss rate decreased. No difference in photosynthesis activity was observed between the control and the MJ-treated seedlings under optimal growth conditions. The impact of cadmium caused a drop in the net photosynthesis activity of the wheat (Table 4). In this case, MJ-treated seedlings experienced less of adecrease in the net photosynthesis rate compared with the untreated plants.

### 3.4. Effect of MJ Pre-Treatment on the FA Profile of Wheat Leaves

In wheat leaves, 13 fatty acids were detected, including 5 saturated and 8 unsaturated FAs. In the control plants, the content of saturated FAs decreased over the seven days (Table 5). Some quality changes were also observed, particularly that pentadecanoic acid (15:0) was not detected on the first day of analysis and the myristic acid (MA, 14:0) was not detected on the third day of the experiment (Table 5). MJ pre-treatment led to a drop in FA content; compared with untreated plants, the total amount of saturated FAs was lower. Additionally, quality changes were found; for instance, in the leaves of MJ-treated plants MA was not detected after 24 h, compared to the control. Under Cd stress, the MA and pentadecanoic acid were not detected in leaves of wheat treated by MJ and without MJ pre-treatment on the first day of the experiment (Table 5). The Cd impact led to an increase in palmitic acid (PA, 16:0) and stearic acid (SA, 18:0) content on the third and seventh days of exposure compared to the control. Additionally, the total amounts of saturated FAs on the third and seventh days were higher than those in the control (Table 5). The MJ-treated plants had similar quantity and quality changes with regard to FA content compared with untreated plants exposed to Cd. Despite the slight increase in PA and SA accumulation, the MJ-treated plants were also characterized by a higher total amount of saturated FAs compared to the control (Table 5).

In the control plants the amount of unsaturated FAs also decreased during the seven days, except 18:3n-3, α-linolenic acid (ALA) (Table 6). During the three days of growing, the control plants had increased ALA content. The total amount of unsaturated FAs in MJ-treated plants was less than in the control. However, during the three days of growing MJ treatment led to an increase in 18:2n-6 (LIN) and ALA FA content in comparison to the control (Table 6). On the third day, the amount of 22:6n-3 FA (DHA) was also higher, resulting in higher total unsaturated FA content compared with the untreated plants (control). The influence of Cd caused an increase in the total amount of unsaturated FAs during the seven days. For instance, under these conditions the wheat leaves contained higher amount of 18:1n-9, 18:2n-6, 18:3n-3, 20:1n-9, 20:5n-3 and 22:6n-3 (Table 6). MJ-treated plants under stress conditions (Cd) experienced similar changes to plants growing under optimal conditions (an increase in LIN and ALA content), but this was less prevalent than under Cd influence alone (Table 6).

## 4. Discussion

Jasmonic acid and its various derivatives are phytohormones that regulate plant growth and development [9,27]. The effect of the exogenous application of jasmonates on plants’ physiological processes is being actively studied at the present time. The stimulating or inhibiting effect of exogenous MJ application depends on its concentration and the duration of exposure. For example, some ripening parameters of plants can be inhibited by high concentrations of exogenous MJ [28], as well as seed germination [29]. In the present study, it was shown that the height of seedlings treated with exogenous MJ was slightly lower compared to untreated plants. A similar effect was also found in tobacco [30], rice [31] and pea [32] plants. However, there are data on the absence of the effect of exogenous MJapplication on the linear dimensions of plants, for example soybean [33], *Catharanthus roseus* [34] and pea [35]. At the same time, the accumulation of plant biomass, in this case, increased compared to untreated plants; this was also observed in wheat in the present study, and not only here (see [36]), as well as in plants including rice [12], *Isatisin digotica* [37] and pea [35].

Currently, less is known about the role of jasmonates in plant resistance to heavy metals [38,39]. The study showed that Cd negatively affects the growth and biomass accumulation of wheat seedlings. A similar effect was found in plants including barley [40,41], rice [42] and sweet sorghum [43], which is largely associated with the negative effect of Cd on cell division and elongation [3], along with an effect on the number of physiological and biochemical processes (in particular, a decrease in the content of pigments and photosynthesis intensity, a change in sulfur metabolism and the rate of nitrogen and phosphorus assimilation, and an increase in lipid peroxidation) that indirectly affects growth [5,44]. In wheat plants pretreated with MJ, Cd also led to a drop in growth, but it was smaller than in the untreated plants. The same effect was observed in rice [12] and *Mentha arvensis* [45] in MJ treatment under Cd exposure. It is well known that the productivity of plants under stress conditions depends on photosynthesis activity, but there is less known about the effect of MJ on photosynthesis under heavy metal exposure. The present study showed the absence of any effect of exogenous MJ application on the rate of net photosynthesis; this corresponds with data obtained on *B. napus* [46], rice [31] and *Amarin* hybrids [47]. It is known that Cd affects the rate of photosynthesis (i.e., there is a decrease). It has been discussed that such results could be associated with changes in the anatomical structure of the leaf, damage tothe chloroplast structure, a decrease in pigment content, and changes in the light and dark reactions of photosynthesis [48,49]. In this study, a positive effect of MJ on the net photosynthesis rate was observed. MJ-treated plants were characterized by a higher net photosynthesis rate than untreated plants exposed to Cd. A similar effect was also described by other authors, in particular in mint plants [45] under Cd, in rice in the presence of high concentrations of arsenic [31] and in rapeseed exposed to salinity [46]. This positive effect of exogenous MJ application can be associated with its promotion of an increase in the content of photosynthetic pigments [50], the efficiency of photosystem II and RUBISCO activity [45] in plants under cadmium stress. Additionally, a decrease in the accumulation of reactive oxygen species was demonstrated in the presence of MJ [38].

In addition, the higher net photosynthesis rate that led to a greater accumulation of biomass by MJ-treated wheat seedlings exposed to Cd, compared to untreated plants, could be a result of the lower Cd ion concentration in plants that was shown in this study, as well as by other authors [12,19,43]. The mechanisms that form the basis of this effect are still unknown. However, it has been shown that MJ stimulates sulfur assimilation and the accumulation of reduced glutathione, which binds cadmium ions, thereby participating in its inactivation [51]. In addition, the promotion effect of MJ on the content of lignin [37] and cellulose [15] in the cell wall was demonstrated. On the one hand, this leads to an increase in the rigidity of the cell wall and a decrease in permeability, and on the other hand, to an increase in metal ion binding sites. Such rearrangements are one of the mechanisms aimed at increasing plant tolerance to heavy metals [52].

The results noted above and thediscussion associated with them correspond to the data on fatty acid content described in the present study. One of the main mechanisms of plant adaptation to different stresses is the qualitative and quantitative rearrangement of fatty acid composition in the membrane [53,54,55]. The study demonstrated the influence of MJ on the FA profile. In particular, MJ pre-treatment caused a decrease in all identified FAs, except ALA, which is one of the key unsaturated FAs of ‘terrestrial plants’. On the third growth day under optimal conditions, MJ application led to an increase in the content of LIN, ALA, PA and DHA, but on seventh day the MJ-promoting effect was alleviated. The higher content of ALA under MJ treatment has been observed previously, in other studies [29,56]. ALA is important for plants; its content in thylakoid galactolipids can reach 95%, which determines its vital metabolic and structural role in chloroplasts [25]. Along with PA and LIN, ALA is a main structural component in the glycophospholipids of mitochondrial membranes [57]. Moreover, ALA is a precursor for n-3 polyunsaturated FAs (PUFA), including DHA, usually called ‘a fatty acid of adaptation’ [52,58]. Considering that exogenous MJ increases the activity of lipoxygenase, the key enzyme of FA synthesis [59], and the content of endogenous MJ [19], it can be supposed that MJ is capable of self-biosynthesis regulation by promoting MJ synthesis de novo or its demethylation. However, this assumption needs further investigation.

MJ pre-treatment caused an increase in the content of PA and DHA that, we assume, indicated a stress-induced change in biomembranes’ rigidity and the ‘intension’ of the transmembrane processes. The metabolites of PA, 18:1n-9 and LIN catabolism were mainly presented in the lipids of tonoplast in the area of microdomains (rafts) [57]. In comparison to saturated FAs, the PUFA as DHA had higher flexibility rates due to its physicochemical and conformational properties. The accumulation of such PUFAs, including DHA, to maintain the biomembrane functionality that resulted in a stable thermodynamic microenvironment for the activity of membrane-bound integral proteins is a universal mechanism for the adaptation of living organisms to stress [58,60,61,62]. Additionally, the synthesis of PUFA, including DHA, is associated with cell proliferation and its control [25]; however, the role of this FA in plant metabolism remains unknown. A rapid decrease in the content of saturated FA due to MA in plants treated by MJ was found. It is known that there were opposite changes in the composition of saturated FAs in mitochondrial membranes: the MA content decreased while the PA content increased. It is supposed that MA acts as a functional substitute [63] due to the similar physico-chemical properties of both FAs. Such reversible transformations can be considered a ‘metabolic swing’, which is a biochemical adaptation mechanism aimed at maintaining homeostasis under changing environmental conditions. In general, the overall decrease in the content of fatty acids in wheat plants upon treatment with MJ is probably associated with their redistribution and involvement in energy-dependent cell processes.

Cd caused an accumulation of 18:2n-6, 18:3n-3 and 22:6n-3 FAs. Under prolonged exposure, the PA content increased. Overall, the saturated and unsaturated FAs in wheat leaves under Cd exposure were higher in comparison to the control. Under Cd exposure, as well as under optimal conditions, the PA content increased along with the absence of MA, suggesting the elongation of MA to PA [64]. In the present study, an increase in the PA amount was shown on the third day, with the Cd ions’ intake by the leaf cells, supporting the stress-induced response. The changes (an increase) in PA in plants under different stress conditions have been reviewed in detail by Zhukov, 2015 [63]. Along with the accumulation of PA, an increase in LIN was also observed. As noted above, these fatty acid components and their derivatives are components of the tonoplast, which is known to play an important role in the isolation of cadmium in the vacuole. At the same time, it should be taken into account that PA is involved in the anchoring of proteins on the inner part of the membrane by binding to cysteine and serine residues [64,65], ensuring the functioning of membrane-bound tonoplast transporter proteins, which is probably one of the mechanisms of the compensatory reaction of plants in the presence of Cd ions. The accumulation of PA and LIN acids under Cd exposure was found in tomato plants [66], corn [67] and *Mesembryanthemum crystallinum* [68]. The increased content of DHA in plants under Cd exposure—as in the case of the action of MJ—is probably associated with its role in the restructuring of the biomembrane (in part, an increase in rigidity against the background of the preservation of individual areas where the functioning of membrane-bound enzymes is maintained within the physiological optimum).

The changes in the quantitative parameters of the FA profile in wheat plants in the presence of cadmium are similar to those under the action of only MJ. In general, under metal stress, the changes are aimed at the thickening of the membrane structure and an increase in the saturation of FAs, which can probably be considered another mechanism of plant resistance to the action of the metal [69]. This can be explained by the fact that the action of Cd itself is aimed at the activation of MJ signaling and synthesis [50,70,71]. It should be pointed out that, in pre-treated MJ plants under Cd exposure, the content was higher than in untreated plants.This was probably due to the key functional role of ALA in plastids [25], and its higher concentration provided the rise in the level of photosynthesis.

In wheat plants exposed to Cd that had been pre-treated with MJ, the changes in the FA content after one day were similar to the action of MJ under optimal conditions, which can be explained by a decrease in the cadmium content. While there werelonger exposures, the FA profile was similar to that under the action of cadmium but less pronounced, which can also be associated with a lower concentration of the metal. Moreover, the composition of the FA profile on day seven was very similar to that of the control, which may indicate the successful action of compensatory reactions and plant tolerance under these conditions. Cumulative effects of two chemical agents (MJ and Cd) were not found.

## 5. Conclusions

Pre-treatment with MJ under optimal growth conditions had no effect on the linear dimensions and net photosynthesis rate of wheat; however, it promoted the accumulation of fresh shoot biomass. Exogenous MJ caused a general decrease in the total fatty acid content, except forα-linolenic, palmitic and docosahexaenoic acids. Under stress conditions (Cd influence), pre-treatment with MJ caused an increase in the netphnet photosynthesisotosynthesis rate and biomass accumulation of seedlings, which is possibly associated with a lower Cd concentration in wheat compared to the untreated plants. The application of MJ to plants under Cd stress did not lead to cumulative effects on the fatty acid content. It is likely that, under the influence of external chemical agents (MJ and Cd), the change in the composition of fatty acids was directed locally (formation of rafts) or towards the integral packing of membranes. The stress-induced increase in palmitic acid, along with the absence of myristic acid, can be considered a biochemical mechanism of adaptation at the lipid metabolism to metal tolerance and/or the presence of an alternative pathway for plant adaptation to chemical stresses (including the maintenance of cellular biomembrane fluidity). However, additional studies are needed to confirm this.

## Figures and Tables

**Table 1:** Effect of MJ on Cd content (µg/g dry weight) in roots and shoots of wheat seedlings.

Plant Organ	Variant	Exposure, h		
		24	72	168
Roots	Cd Cd + MJ	199.00 ± 3.89 e	643.50 ± 18.59 c	1075.25 ± 30.49 a
		137.00 ± 3.29 f	406.25 ± 4.57 d	836.75 ± 18.06 b
Leaves	Cd Cd + MJ	2.65 ± 0.27 ij	4.76 ± 0.32 h	6.09 ± 0.51 g
		1.37 ± 0.25 k	2.11 ± 0.18 j	3.57 ± 0.40 i

Values are means ± SE (*n* = 3). Different letters indicate significant differences between treatments (*p <* 0.05), determined by Fisher’s least significant difference (LSD) test.The Cd concentration in plants prior to exposure to MJ and Cd was 0.01 ± 0.01 mg/g dry weight. The Cd content in control plants independend on the MJ application being below the limit of quantification.

**Table 2:** Effect of MJ on height (cm) of wheat seedlings.

Variant	Exposure, h			
	0	24	72	168
Control	18.64 ± 0.10 f	21.06 ± 0.18 de	23.69 ± 0.28 c	34.71 ± 0.42 a
Control + MJ	18.33 ± 0.09 f	20.09 ± 0.18 e	21.84 ± 0.24 d	32.49 ± 0.44 b
Cd	18.64 ± 0.10 f	20.68 ± 0.15 e	21.62 ± 0.25 d	25.11 ± 0.44 c
Cd + MJ	18.33 ± 0.09 f	19.98 ± 0.15 e	20.53 ± 0.17 e	23.01 ± 0.33 c

Values are means ± SE (*n* = 45). Different letters indicate significant differences between treatments (*p <* 0.05), determined by Fisher’s least significant difference (LSD) test.

**Table 3:** Effect of MJ on fresh weight (g) of wheat seedlings.

Variant	Exposure, h			
	0	24	72	168
Control	153.11 ± 3.44 j	191.88 ± 5.81 f	236.96 ± 14.02 c	334.11 ± 12.30 b
Control + MJ	161.27 ± 3.71 i	183.01 ± 4.40 g	245.46 ± 11.95 c	388.71 ± 12.88 a
Cd	153.11 ± 3.44 j	174.06 ± 3.12 h	195.06 ± 4.80 f	216.03 ± 3.08 d
Cd + MJ	161.27 ± 3.71 i	180.17 ± 4.33 g	202.22 ± 4.81 e	224.84 ± 2.83 c

Values are means ± SE (*n* = 30). Different letters indicate significant differences between treatments (*p <* 0.05), determined by Fisher’s least significant difference (LSD) test.

**Table 4:** Effect of MJ on net photosynthesiss rate (µmol CO_2_ m^−1^s^−1^) of wheat seedlings.

Variant	Exposure, h			
	0	24	72	168
Control	3.27 ± 0.10 b	3.67 ± 0.06 a	3.18 ± 0.03 c	2.24 ± 0.09 e
Control + MJ	3.30 ± 0.09 b	3.66 ± 0.08 a	3.33 ± 0.05 b	2.36 ± 0.09 e
Cd	3.27 ± 0.10 b	2.16 ± 0.07 de	1.95 ± 0.08 e	0.64 ± 0.06 g
Cd + MJ	3.30 ± 0.09 b	2.26 ± 0.08 d	2.22 ± 0.08 d	0.91 ± 0.06 f

Values are means ± SE (*n* = 15). Different letters indicate significant differences between treatments (*p <* 0.05), determined by Fisher’s least significant difference (LSD) test.

**Table 5:** Effect of exogenous MJ on the content of saturated FAs (mkl/mL) in wheat leaves under Cd stress.

Variant of Exposure and Duration (h)	Myristic C 14:0	Pentadecanoic C 15:0	Palmitic C 16:0	Margaric C 17:0	Stearic C 18:0	Total
Control						
0	25.44	0.80	221.93	3.47	44.53	296.17
24	1.13	0.00	90.65	1.01	20.51	113.3
72	0.00	0.00	32.84	0.30	11.06	44.20
168	0.00	0.00	38.30	0.41	12.51	51.22
Control + MJ						
0	24.29	0.16	201.00	2.64	38.05	266.14
24	0.00	0.00	58.26	0.51	17.81	76.58
72	0.00	0.00	87.88	0.60	24.17	112.65
168	0.00	0.00	38.47	0.32	12.97	51.76
Cd						
0	25.44	0.80	221.93	3.47	44.53	296.17
24	0.00	0.00	90.62	0.89	20.80	112.31
72	0.00	0.00	83.65	0.66	23.01	107.32
168	0.00	0.00	69.08	0.49	18.90	88.47
Cd + MJ						
0	24.29	0.16	201.00	2.64	38.05	266.14
24	0.00	0.00	44.17	0.44	12.95	57.56
72	0.00	0.00	61.92	0.36	17.37	79.65
168	0.00	0.00	55.27	0.42	15.56	71.25

**Table 6:** Effect of exogenous MJ on the content of unsaturated FAs (mkl/mL) in wheat leaves under Cd stress.

Variant of Exposure and Duration (h)	Hexadecenoic C 16:1n-7	Oleic C 18:1n-9	Linoleic C 18:2n-6	Linolenic C 18:3n-3	Eicosenoic C 20:1n-9	Eicosadienoic C 20:2n-6	Eicosapentaenoic C 20:5n-3	Docosahexaenoic C 22:6n-3	Total
Control									
0	91.24	285.88	30.95	94.60	10.42	5.28	9.12	29.8	557.29
24	18.55	64.77	18.34	116.72	5.12	3.56	2.13	6.72	235.91
72	1.41	6.21	15.57	123.89	1.78	3.16	0.81	2.53	178.55
168	2.11	9.55	16.03	66.29	1.84	3.17	1.12	3.77	103.88
Control + MJ									
0	61.80	234.59	30.83	88.90	12.34	4.84	7.55	24.30	465.15
24	3.34	12.70	19.96	181.94	2.12	3.25	0.92	2.80	227.03
72	6.66	27.10	25.76	161.63	2.61	3.40	2.17	7.82	237.15
168	0.62	3.37	15.25	48.12	1.63	3.1	0.73	2.84	75.66
Cd									
0	91.24	285.88	30.95	94.60	10.42	5.28	9.12	29.8	557.29
24	14.58	46.63	23.62	166.71	4.30	3.54	2.55	8.77	270.70
72	7.95	27.06	22.26	122.27	2.94	3.39	1.95	6.79	229.62
168	2.24	14.62	25.00	58.56	1.91	3.21	1.14	3.74	110.42
Cd + MJ									
0	61.80	234.59	30.83	88.90	12.34	4.84	7.55	24.30	465.15
24	3.49	12.27	18.20	134.39	2.21	3.22	1.02	3.23	178.03
72	1.43	6.40	21.97	99.62	1.79	3.25	0.81	2.52	137.78
168	2.04	17.68	22.86	35.18	1.91	3.17	0.98	3.10	86.92

## Data Availability

All data and tables in this manuscript are original.
